# Radiofrequency Ablation of Gasserian Ganglion in Metastatic Ewing Sarcoma

**DOI:** 10.7759/cureus.44675

**Published:** 2023-09-04

**Authors:** Volodymyr Toropchyn, Sanjeev Kumar, Dawei Guan

**Affiliations:** 1 Anesthesiology, University of Florida, Gainesville, USA; 2 Anesthesiology/Pain Medicine, University of Florida, Gainesville, USA; 3 Surgery, University of Florida, Gainesville, USA

**Keywords:** trigeminal nerve, facial pain, radiofrequency ablation, jaw cancer, gasserian ganglion

## Abstract

We report the case of a 28-year-old male patient diagnosed with extensive metastatic Ewing sarcoma that affected his facial bones. The patient came to us with left-sided jaw/facial pain that could not be managed successfully through conservative methods. We utilized radiofrequency ablation (RFA) of the left Gasserian (trigeminal) ganglion to alleviate the patient's pain successfully. Although this method is commonly used to treat trigeminal neuralgia, to the best of our knowledge, this is the first documented case where Gasserian ganglion RFA has been successfully used to manage pain in a patient with jaw cancer.

## Introduction

Radiofrequency ablation (RFA) is a minimally invasive technique that employs thermal energy to eliminate parts of the trigeminal nerve ganglion responsible for transmitting pain signals. Studies have shown that RFA provides enduring relief from pain in patients with resistant trigeminal neuralgia, all with a low rate of complications [1].

Gasserian Ganglion RFA is commonly used to treat severe trigeminal neuralgia [2,3]. This condition is characterized by sudden, short-lived bouts of stabbing or sharp pain, typically on one side of the face. The pain arises from the trigeminal nerve root compression, often just millimeters from its connection with the pons [4]. While such compression is frequently attributed to anomalous vascular loops (most commonly those of the superior cerebellar artery), it can also result from tumors at the cerebellopontine angle or arteriovenous malformations [5].

While Gasserian Ganglion RFA is highly effective for severe cases of trigeminal neuralgia, there is limited evidence of the efficacy of this technique for chronic jaw pain due to a tumor that does not directly compress a trigeminal nerve root.

We present the case of a young man diagnosed with Ewing sarcoma of the clavicle with metastatic involvement of the facial bones. The patient presented with severe pain in left trigeminal nerve distribution (mainly V3 with a minor component of V2), refractory to conservative management. In total, we performed three radiofrequency ablation sessions of the Gasserian ganglion, which resulted in significant and long-lasting pain relief. The patient's quality of life was markedly improved every time with minimal side effects.

## Case presentation

A 28-year-old male was initially referred to our clinic with chronic jaw pain. The pain was predominantly located in the left jaw at the V3 distribution. Fourteen years prior, the patient was diagnosed with Ewing Sarcoma of the clavicle, which subsequently metastasized to the mandible, pelvis, and sphenoid, causing jaw pain. At the time of the consultation, he was undergoing chemotherapy every four weeks and had completed his radiation therapy.

The patient described the pain as throbbing and tingling, akin to electric shocks, shooting, and stabbing. It was constant, gradually started, and intensified over time. He rated the pain as 10/10 at its worst and, on average, 4/10. The pain was exacerbated by chewing, hot fluids, hot weather, touch, light exercises, prolonged activity, and talking. Rest, medication, distractions, and cold food alleviated it. The pain caused significant stress in his life, impacting his general activity, ability to perform tasks at home or work, sleep, and overall enjoyment of life.

**Figure 1:**
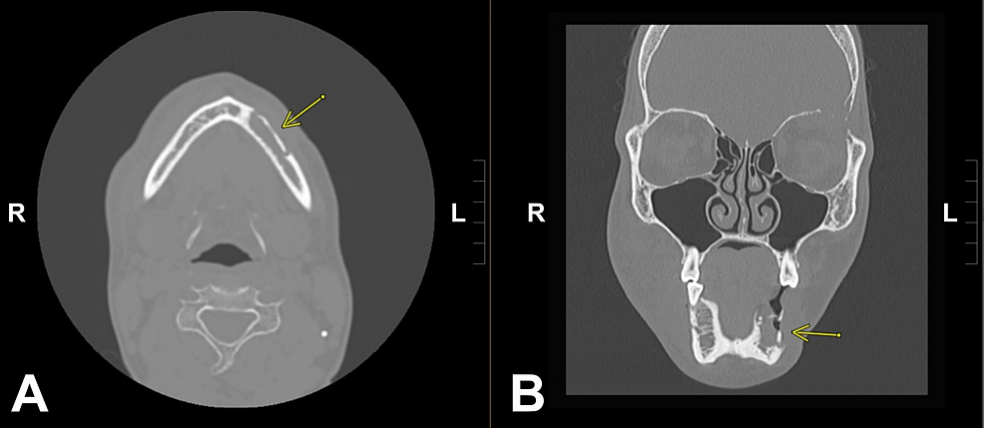
Axial (A) and Frontal (B) CT scan images demonstrating a metastatic lesion to the left hemimandible (yellow arrows).

**Figure 2:**
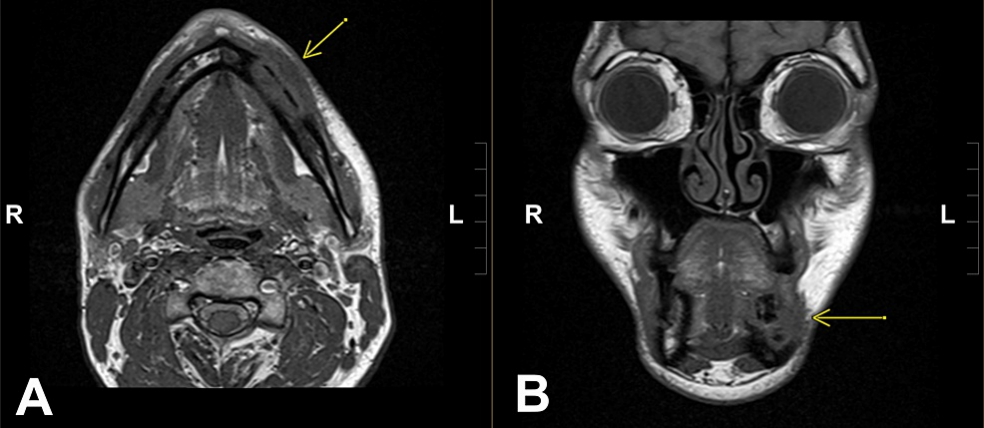
The Ewing sarcoma metastatic lesion demonstrated on Axial (A) and Frontal (B) MRI images as a lytic lesion in the anterior left mandibular body (yellow arrows).

He had previously attempted conservative management, including medical therapy with gabapentin, acetaminophen, cyclobenzaprine, hydromorphone, methadone, ketorolac, and rest. Unfortunately, these interventions failed to provide the desired pain relief (Figure 1-2).

**Figure 3:**
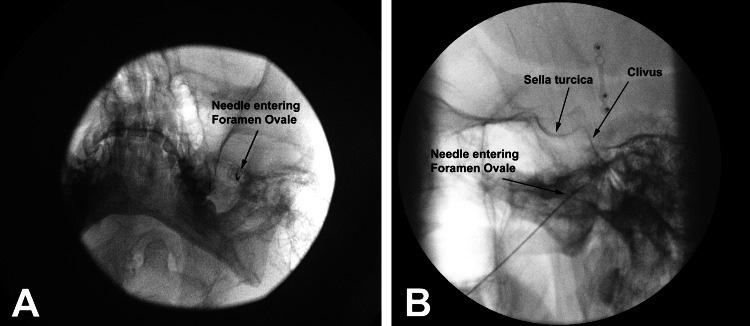
Submental View (A) and Lateral View (B) of the needle entering Foramen Ovale.

The patient agreed to proceed with Gasserian ganglion Radiofrequency Ablation (RFA). We performed the procedure using the following technique (Figure 3):

The foramen ovale was identified with the help of a steep submental tilt of the C-arm image intensifier, and a 10 cm, 22-gauge radiofrequency cannula with a curved 5 mm active tip was carefully guided to the lateral edge of the foramen. Once inside the Meckel's cave, the cannula was guided to the mandibular division of the Gasserian ganglion about 10 mm below the spheno-clival line. Motor stimulation was performed for the third trigeminal nerve (V3) division, and the masseter contraction was seen at 0.3 mv. Four thermal radiofrequency lesions were created for 60 seconds each, starting at 55 degrees Celsius with 5 5-degree increments every minute till 70 degrees. The needle was then rotated medially and advanced cephalad to reach the approximate location of the second division of the gasserian ganglion at a location 5 mm below the spheno-clival line. Sensory stimulation at 0.4mv stimulated the second trigeminal nerve (V2) division and concordant pain in the V2 distribution. One thermal lesion was created here at 70 degrees Celsius for 60 seconds. The needle and cannula were removed upon completion, and a band-aid was applied to the insertion point. 

Following the procedure, the patient experienced an 80% reduction in left-sided jaw and facial pain that persisted for approximately four months. He subsequently requested further ablation treatments and, to date, has undergone two more left Gasserian ganglion RFAs. With these additional procedures, the patient reported comparable pain relief lasting about 6 months each time. The patient's quality of life was markedly improved, with minimal side effects after each procedure.

## Discussion

The trigeminal nerve (Cranial Nerve 5) is the main nerve responsible for transmitting sensory information from the face and head to the brain, doing so through its three divisions. The Gasserian ganglion is a collection of nerve cell bodies where the three divisions of the trigeminal nerve converge before they enter the brainstem [6]. Hence, it constitutes a good target for an ablation procedure in patients with pain in the trigeminal nerve distribution. As suggested by its name, the trigeminal nerve has three divisions: V1 (Ophthalmic Branch), V2 (Maxillary Branch), and V3 (Mandibular Branch). All of these can potentially be targeted with Gasserian Ganglion RFA. However, the branches most often selected for treatment are V2 and V3. The V1 branch is targeted less frequently, as it may lead to corneal anesthesia. This, in turn, can result in corneal abrasions, scarring, and blindness.

We have described a case of effective treatment of cancer-related pain in a patient with metastatic jaw cancer utilizing Gasserian ganglion RFA. Our experiences have led us to perform the Gasserian Ganglion/Cistern ablation in certain cancer patients who suffer from severe pain due to cancer involvement in these areas. While this procedure is traditionally indicated for trigeminal neuralgia [2,3] - and we routinely perform it for such cases - we believe it to be invaluable for some patients with jaw cancer, who continue to experience severe pain even after undergoing radiation and surgery. In this case, we have demonstrated that this procedure can effectively manage pain, improving the patient's ability to eat and drink more comfortably. We believe that the ablation of the Gasserian Ganglion/Cistern can effectively control pain originating within the jaw sclerotome. In patients with extensive cancer who have already undergone radiation and/or surgery, the performance of this procedure becomes more challenging due to the presence of fibrosis and scar tissue. Nevertheless, the potential to provide these patients with several months of pain relief and an improved quality of life is highly rewarding.

## Conclusions

To our knowledge, no existing literature addresses the use of this procedure specifically for cancer pain not caused by direct compression of the trigeminal nerve root. The presented case underscores the need for further research to confirm the effectiveness and safety of this procedure. It is also essential to develop approaches to anatomical challenges posed by post-radiation fibrosis and prior surgeries in patients with cancer-related pain.
